# Risk Factors and Scoring Systems to Predict the Mortality Risk of Afebrile Adult Patients with Monomicrobial Gram-Negative Bacteremia: A 10-Year Observational Study in the Emergency Department

**DOI:** 10.3390/diagnostics14090869

**Published:** 2024-04-23

**Authors:** Chung-Pang Wang, Ming-Shun Hsieh, Sung-Yuan Hu, Shih-Che Huang, Che-An Tsai, Chia-Hui Shen

**Affiliations:** 1 Department of Emergency Medicine, Taichung Veterans General Hospital, Taichung 40705, Taiwan; cpw0509@gmail.com (C.-P.W.); aa1239076@gmail.com (C.-H.S.); 2 Department of Emergency Medicine, Taipei Veterans General Hospital, Taoyuan Branch, Taoyuan 330, Taiwan; edmingshun@gmail.com; 3 Department of Emergency Medicine, Taipei Veterans General Hospital, Taipei 11217, Taiwan; 4 School of Medicine, National Yang Ming Chiao Tung University, Taipei 11217, Taiwan; 5 Department of Post-Baccalaureate Medicine, College of Medicine, National Chung Hsing University, Taichung 40201, Taiwan; 6 Institute of Medicine, Chung Shan Medical University, Taichung 40201, Taiwan; 7 School of Medicine, Chung Shan Medical University, Taichung 40201, Taiwan; cucu0214@gmail.com; 8 Department of Emergency Medicine, Chung Shan Medical University Hospital, Taichung 40201, Taiwan; 9 Lung Cancer Research Center, Chung Shan Medical University Hospital, Taichung 40201, Taiwan; 10 Division of Infectious Disease, Department of Internal Medicine, Taichung Veterans General Hospital, Taichung 40705, Taiwan; lucky-sam@yahoo.com.tw

**Keywords:** afebrile, gram-negative bacteremia, monomicrobial, mortality, scoring system

## Abstract

Background: The mortality rate of afebrile bacteremia has been reported to be as high as 45%. This investigation focused on the risk factors and predictive performance of scoring systems for the clinical outcomes of afebrile patients with monomicrobial gram-negative bacteria (GNB) in the emergency department (ED). Methods: We conducted a retrospective analysis of afebrile adult ED patients with monomicrobial GNB bacteremia from January 2012 to December 2021. We dissected the demographics, clinical pictures, and laboratory investigations. We applied five scoring systems and three revised systems to predict the clinical outcomes. Results: There were 600 patients included (358 males and 242 females), with a mean age of 69.6 ± 15.4 years. The overall mortality rate was 50.17%, reaching 68.52% (74/108) in cirrhotic patients. *Escherichia coli* was the leading pathogen (42.83%). The non-survivors had higher scores of the original MEDS (*p* < 0.001), NEWS (*p* < 0.001), MEWS (*p* < 0.001), qSOFA (*p* < 0.001), and REMS (*p* = 0.030). In univariate logistic regression analyses, several risk factors had a higher odds ratio (OR) for mortality, including liver cirrhosis (OR 2.541, *p* < 0.001), malignancy (OR 2.259, *p* < 0.001), septic shock (OR 2.077, *p* = 0.002), and male gender (OR 0.535, *p* < 0.001). The MEDS demonstrated that the best predictive power with the maximum area under the curve (AUC) was measured at 0.773 at the cut-off point of 11. The AUCs of the original NEWS, MEWS, qSOFA, and REMS were 0.663, 0.584, 0.572, and 0.553, respectively. We revised the original MEDS, NEWS, and qSOFA by adding red cell distribution width, albumin, and lactate scores and found a better predictive power of the AUC of 0.797, 0.719, and 0.694 on the revised MEDS ≥11, revised qSOFA ≥ 3, and revised NEWS ≥ 6, respectively. Conclusions: The original MEDS, revised MEDS, revised qSOFA, and revised NEWS were valuable tools for predicting the mortality risk in afebrile patients with monomicrobial GNB bacteremia. We suggested that clinicians should explore patients with the risk factors mentioned above for possible severe infection, even in the absence of fever and initiate hemodynamic support and early adequate antibiotic therapy in patients with higher scores of the original MEDS (≥11), revised MEDS (≥11), revised NEWS (≥6), and revised qSOFA (≥3).

## 1. Introduction

The criteria of systemic inflammatory response syndrome (SIRS) are an easy-to-apply set of clinical parameters that aid clinicians in identifying potential patients with systemic infection [1,2,3]. Clinicians frequently rely on fever as a criterion for initiating infection surveys in the emergency department (ED). Patients with fever accompanied by signs of severe infection, such as a change in mental status or low blood pressure, can aid physicians in tentatively diagnosing bacteremia [4]. However, patients with bacteremia might present to the ED without evidence of fever. Delays in diagnosis and treatment might occur in these patients. Previous studies have identified that afebrile bacteremia has a distinct presentation in the elderly or patients with an immunosuppressed status [5,6,7,8,9]. According to a previous retrospective cohort study, the authors found that 14.9% (140/937) of patients with episodes of afebrile bacteremia during the one-year study period had a high mortality rate reaching 45% [9]. In a study, Lin et al. revealed that age > 64 years, liver cirrhosis, malignancy, use of alcohol, polymicrobial bacteremia, anemia, and sepsis were independent positive predictors of short-term mortality in ED bacteremic patients [10]. In a case–control study, the ED patients with polymicrobial bacteremia had a higher mortality rate than those with monomicrobial bacteremia [11]. Several studies reported that the cause of higher mortality rates partly attributed to afebrile bacteremia with lowered level warnings by the clinicians. This cause may be related to a delayed or absent response to antimicrobial therapy in many patients within 24 h of admission [12,13,14,15]. Another possibility for a terrible prognosis in patients with afebrile bacteremia is the development of a poor immune response to bacterial invasion, putting the patient at risk of impediment and death [16].

In the literature review, there is no research on afebrile bacteremia on specific factitive microorganisms to assess the association of different pathogens on clinical outcomes. In this study, we analyzed the clinical characteristics and laboratory data of 600 afebrile adult patients visiting the ED with bacteremia caused by monomicrobial gram-negative bacteria (GNB) during a 10-year study period. Our goal is to clarify specific risk factors and various scoring systems, in order to predict the mortality risk of bacteremia caused by monomicrobial GNB in afebrile adult patients.

## 2. Materials and Methods

### 2.1. Data Collection and Definition

The institutional review board of Taichung Veterans General Hospital (TCVGH) approved this study (No. CE23126B). We retrospectively analyzed afebrile patients with monomicrobial GNB, confirmed by a blood culture collected in the ED. Patients’ data were extracted from the electronic medical records of TCVGH for ten years, from January 2012 to December 2021.

We included patients aged 18 years and older in this study. We determined the afebrile status as no evidence of fever history (body temperature less than 37.8 °C) and measured fever within the first 48 h through the ED course. We defined bacteremia of monomicrobial GNB as a positive result of at least one set of a single gram-negative microorganism. Patients with polymicrobial bacteremia, in which more than one gram-negative microorganism and gram-negative bacteremia mixed infection with gram-positive or anaerobic species, were excluded from this study.

The following data were collected: demographics, preexisting comorbid conditions, vital signs, laboratory findings, and microorganisms isolated from the blood cultures. During the patients’ ED course, vital signs were collected, including initial and serial values from the nursing records. We defined the primary outcome as the rate of overall in-hospital mortality. We performed univariate and multivariate analyses to evaluate the mortality risk. In addition, we used five scoring systems to predict the clinical outcomes. We found positive laboratory predictors related to the mortality risk, and then we revised three of five scoring systems by adding scores to refine their risk-predicting performance.

### 2.2. Scoring Systems

The clinical scoring systems in this study included the Mortality in Emergency Department Sepsis (MEDS) Score, National Early Warning Score (NEWS), Modified Early Warning Score (MEWS), quick Sequential Organ Failure Assessment (qSOFA), and Rapid Emergency Medicine Score (REMS). MEWS contained six parameters: body temperature, heart rate, systolic blood pressure, respiratory rate, and the alert, verbal, painful, unresponsive (APVU) scale. REMS evaluated six variables: age, mean arterial pressure, heart rate, respiratory rate, O_2_ saturation, and Glasgow Coma Scale (GCS). We used these scoring systems to analyze the clinical outcomes and mortality risk. We disclosed indicators in the abovementioned scoring systems in Table S1 (Supplementary Materials).

### 2.3. Statistic Analysis

We described and compared the characteristics of the survivors and non-survivors. Continuous data were presented as mean ± standard deviation (SD); whereas, we presented categorical variables as counts and percentages. For univariate analysis, we used the chi-squared tests or Fisher’s exact test to compare categorical variables and Mann–Whitney tests to compare continuous data regarding the mortality risk in the survivors and non-survivors. We conducted univariate and multivariate analyses using the logistic regression model to assess the potential predictors for mortality, and we presented the results as odds ratio (OR) and confidence interval (CI). We performed the Kaplan–Meier survival analysis to examine the survival differences between the survivors and non-survivors. In addition, we used the area under the curve (AUC) of the receiver operating characteristic curve (ROC) to compare different scoring systems for predicting mortality in adult patients with afebrile bacteremia of monomicrobial GNB. We used the cut-off points of scores to stratify the mortality risks regarding sensitivity, specificity, negative predictive value (NPV), and positive predictive value (PPV). A *p*-value < 0.05 was considered statistically significant. We performed all analyses using the Statistical Package for the Social Science (IBM SPSS version 22.0; International Business Machines Corp., New York, NY, USA).

## 3. Results

### 3.1. Demographics, Clinical Characteristics, Primary Outcome, and Comorbidities

During the 10-year study period, we collected data on 743 patients with afebrile bacteremia of GNB. We excluded 58 patients with polymicrobial GNB and 85 patients simultaneously with gram-negative and other bacteremia. Finally, in this study, we enrolled 600 patients with afebrile bacteremia of monomicrobial GNB. We summarized the demographics and clinical findings of 600 patients, including 358 males (59.66%) and 242 females (40.33%) in Table 1. The mean age was 69.59 ± 15.38 years. Adult patients were aged 18–64 (*n* = 220, 36.66%) and 65–74 (*n* = 124, 20.66%). Most patients were aged ≥75 years (*n* = 256, 42.66%). In total, 357 (59.5%) patients had a “Do Not Resuscitate” (DNR) order because of advanced or terminal illness. The non-survivors had a higher rate of having DNR orders (30.1% vs. 88.7%, *p* < 0.001). Malignancy (*n* = 323, 53.83%) was the most common comorbidity. The comorbid conditions were gastrointestinal (GI) disease (*n* = 272, 45.33%), chronic kidney disease (CKD) (*n* = 195, 32.50%), diabetes mellitus (DM) (*n* = 181, 30.16%), hyperlipidemia (*n* = 158, 26.33%), biliary tract disease (*n* = 133, 22.16%), chronic obstructive pulmonary disease (COPD) (*n* = 114, 19%), and liver cirrhosis (*n* = 108, 18%). We found a lower incidence of hyperlipidemia in the non-survivors (57/158, 18.93%) than in the survivors (101/158, 33.77%) (*p* < 0.001).

### 3.2. Laboratory Data

We summarized the laboratory results and scoring systems in Table 2 and Table 3. The non-survivors had lower values of hemoglobin (10.45 ± 2.49 vs. 11.28 ± 2.65, *p* < 0.001), platelet (156.70 ± 112.75 vs. 194.01 ± 119.91, *p* < 0.001), pH (7.34 ± 0.12 vs. 7.39 ± 0.09, *p* = 0.013), and albumin (2.56 ± 0.60 vs. 2.83 ± 0.61, *p* < 0.001). Levels of red cell distribution width (RDW) (17.12 ± 3.18 vs. 15.31 ± 2.63, *p* < 0.001), blood urea nitrogen (BUN) (60.61 ± 44.52 vs. 48.41 ± 38.91, *p* < 0.001), creatinine (Cr) (2.81 ± 2.28 vs. 2.55 ± 2.36, *p* = 0.026), BUN/Cr ratio (26.36 ± 15.94 vs. 22.76 ± 14.49, *p* = 0.001), total bilirubin (3.66 ± 6.45 vs. 1.81 ± 3.16, *p* < 0.001), and lactate (42.82 ± 36.99 vs. 28.24 ± 28.24, *p* < 0.001) were significantly higher in the non-survivors. In addition, prothrombin time (PT) (12.16 ± 2.79 vs. 15.30 ± 7.63, *p* < 0.001) and activated partial thromboplastin time (APTT) (31.60 ± 7.21 vs. 38.89 ± 16.63, *p* < 0.001) showed significant differences between the survivors and non-survivors. The non-survivors had significantly higher scores of the original MEDS (12.63 ± 3.77 vs. 7.92 ± 4.97, *p* < 0.001).The non-survivors had higher scores of the original MEWS (2.31 ± 2.33 vs. 1.64 ± 1.97, *p* < 0.001), NEWS (5.54 ± 3.18 vs. 4.11 ± 3.07, *p* < 0.001), qSOFA (0.76 ± 0.79 vs. 0.55 ± 0.73, *p* < 0.001), and REMS (6.68 ± 3.07 vs. 6.07 ± 2.67, *p* = 0.030) than those of the survivors (Table 3).

### 3.3. Microbiology of MonomicrobialGNB in Patients with Afebrile Bacteremia

Table 4 reveals no significant differences in causative gram-negative pathogens between the survivors and the non-survivors. *Escherichia coli* (*E. coli*) was the leading gram-negative pathogen (*n* = 257, 42.83%). *Klebsiella* spp. (*n* = 120, 20%) were the second most common of the pathogens. Other gram-negative species included *Salmonella* spp. (*n* = 36, 6%), *Pseudomonas* spp. (*n* = 30, 5%), and *Proteus* spp. (*n* = 27, 4.5%).

### 3.4. Outcome and Survival

The overall in-hospital mortality rate of patients with afebrile bacteremia of monomicrobial GNB was 50.17% (301/600). In cirrhotic patients with afebrile bacteremia, the mortality rate even reached 68.52% (74/108). For patients with afebrile bacteremia of monomicrobial GNB, males had significantly higher in-hospital mortality rates than females (67.10% vs. 32.89%, *p* < 0.001). The non-survivors had a higher rate of septic shock (19.93% vs. 10.70%, *p* = 0.002) (Table 1). The non-survivors had a higher incidence of using vasopressors than the survivors (60.46% vs. 37.12%, *p* < 0.001). The survivors had a longer length of hospital stay (LOS) than the non-survivors (22.56 days vs. 16.00 days, *p* < 0.001).

### 3.5. Univariate and Multivariate Analyses to Evaluate the Mortality Risks

We conducted univariate analyses to predict predisposing risk factors of the clinical outcomes of patients with afebrile bacteremia of monomicrobial GNB, and the OR was in Table 5. We found liver cirrhosis (OR 2.541, *p* < 0.001), malignancy (OR 2.259, *p* < 0.001), and septic shock (OR 2.077, *p* = 0.002) were associated with a higher mortality rate. In contrast, females (OR 0.535, *p* < 0.001) and hyperlipidemia (OR 0.458, *p* < 0.001) were associated with a lower mortality rate.

Regarding laboratory data, we found higher levels of RDW (OR 1.260, *p* < 0.001), total bilirubin (OR 1.095, *p* < 0.001), PT (OR 1.273, *p* < 0.001), APTT (OR 1.068, *p* < 0.001), and lactate (OR 1.010, *p* < 0.001) were associated with a higher mortality rate. Moreover, lower levels of pH (OR 0.062, *p* = 0.003), hemoglobin (OR 0.881, *p* < 0.001), platelet (OR 0.997, *p* < 0.001), and albumin (OR 0.468, *p* < 0.001) were significantly associated with a high risk of death.

Regarding the scoring systems, we demonstrated that the higher scores of the original MEDS (OR 1.264, *p* < 0.001), NEWS (OR 1.160, *p* < 0.001), MEWS (OR 1.159, *p* < 0.001), qSOFA (OR 1.430, *p* = 0.001), and REMS (OR 1.079, *p* = 0.009) were associated with a higher mortality rate. We summarized the univariate and multivariate logistic regression analyses of laboratory variables for afebrile patients with bacteremia of monomicrobial GNB (Table 6). The multivariate logistic analyses showed that higher levels of RDW (OR 1.194, *p* < 0.001) and lactate (OR 1.009, *p* = 0.021) were associated with a higher mortality rate. Moreover, lower levels of albumin (OR 0.643, *p* = 0.022) were significantly associated with a high death risk.

### 3.6. Receiver Operation Characteristic (ROC) of Scoring Systems

**Figure 1:**
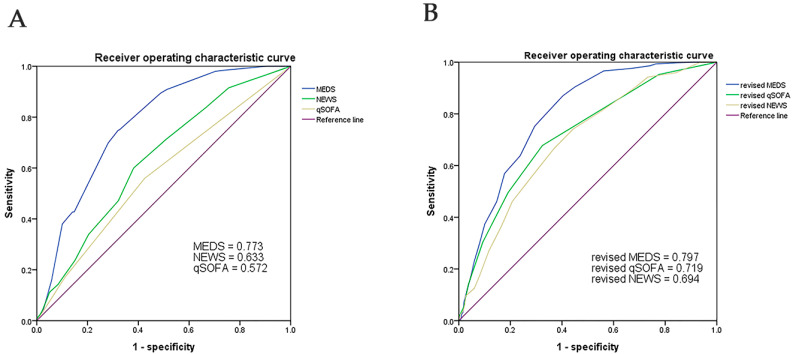
The AUC of the ROC of the original MEDS, NEWS, and qSOFA showed 0.773, 0.633, and 0.572 to predict the mortality risk of patients with afebrile bacteremia of monomicrobial GNB (Panel **A**). The AUC of the ROC for the revised MEDS, qSOFA, and NEWS indicated 0.797, 0.719, and 0.694 to predict the mortality risk of patients with afebrile bacteremia of monomicrobial GNB (Panel **B**). AUC, Area under the curve; MEDS, Mortality in Emergency Department Sepsis; NEWS, National Early Warning Score; qSOFA quick Sequential Organ Failure Assessment; and ROC, receiver operating characteristic curve.

We analyzed the ROC of the original MEDS, NEWS, and qSOFA for accuracy in predicting the mortality risk in patients with afebrile bacteremia caused by monomicrobial GNB (Figure 1 and Table 7). The cut-off point of the original MEDS was 11, with the AUC measured at 0.773 (sensitivity of 75% and specificity of 68%, *p* < 0.001). The cut-off points of the original NEWS and qSOFA were 5 and 1, with the AUC of the ROC measured to 0.633 (sensitivity of 61% and specificity of 62%, *p* < 0.001) and 0.572 (sensitivity of 57% and specificity of 58%, *p* = 0.002), respectively. The original MEDS score best predicted the mortality risk of adult patients with afebrile bacteremia of monomicrobial GNB.

We applied the Youden index to determine RDW, albumin, and lactate cut-off points. We assigned each predictive a score of 1 or 2, relying on the OR of the dichotomized variables. Patients gained additional scores in the original MEDS, qSOFA, and NEWS: if RDW > 16.5%, score 1; if RDW > 19.9%, score 2; if albumin < 3.0 g/dL, score 1; if albumin < 2.4 g/dL, score 2; if lactate > 21 mg/dL, score 1; and if lactate > 48 mg/dL, score 2. We revised the original MDS, NEWS, and qSOFA to refine performance and predict the mortality risk. Figure 1 and Table 8 reveal the results of the revised systems. The AUCs of the ROC of the revised MEDS, revised NEWS, and revised qSOFA were0.797 (sensitivity of 87%, specificity of 60%, *p* < 0.001), 0.719 (sensitivity of 67, specificity of 68%, *p* < 0.001), and 0.694 (sensitivity of 74%, specificity of 56%, *p* < 0.001), respectively. The revised systems demonstrated superiority in predictive performance compared with the original ones.

### 3.7. Kaplan–Meier Survival Curveofthe Original and Revised Scoring Systems

**Figure 2:**
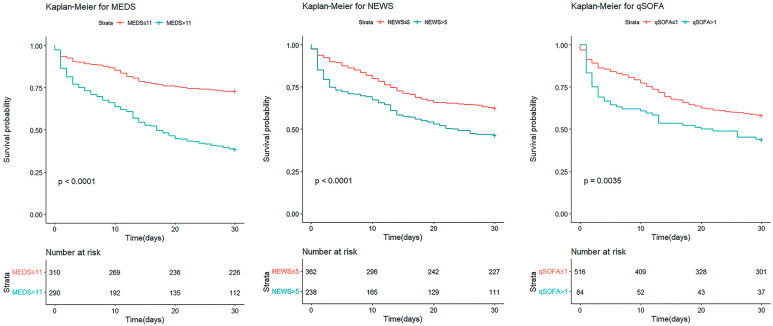
The 30-day cumulative survival rates of patients with afebrile bacteremia caused by monomicrobial GNB were calculated by Kaplan–Meier analyses. The original MEDS, NEWS, and qSOFA cut-off points were 11, 5, and 1, respectively. MEDS, Mortality in Emergency Department Sepsis; NEWS, National Early Warning Score; and qSOFA, quick Sequential Organ Failure Assessment.

**Figure 3:**
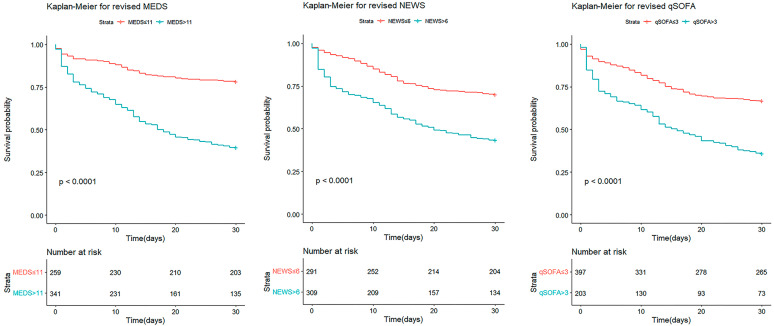
The 30-day cumulative survival rates of patients with afebrile bacteremia caused by monomicrobial GNB were calculated by Kaplan–Meier analyses. The cut-off points of the revised MEDS, revised NEWS, and revised qSOFA were 11, 6, and 3, respectively. MEDS, Mortality in Emergency Department Sepsis; NEWS, National Early Warning Score; and qSOFA, quick Sequential Organ Failure Assessment.

We conducted Kaplan–Meier survival analyses to predict the 30-day cumulative survival rates of patients with afebrile bacteremia caused by monomicrobial GNB. The cut-off points of the original MEDS, NEWS, and qSOFA were 11, 5, and 1, with significant differences (*p* < 0.001, *p* < 0.001, and *p* = 0.002), respectively (Figure 2). The cut-off points of the revised MEDS, NEWS, and qSOFA were 11, 6, and 3, with significant differences (*p* < 0.001, *p* < 0.001, and *p* < 0.001), respectively (Figure 3). The revised scoring systems demonstrated a better predictive performance than the original ones.

## 4. Discussion

The main findings of this study were as follows: (1) Age ≥ 75 years and malignancy were the comorbid conditions most associated with afebrile monomicrobial GNB; (2) The mortality rate in ED patients with afebrile bacteremia caused by monomicrobial GNB was as high as 50.17% and, exceptionally, reached 68.52% in patients with liver cirrhosis; (3) The gram-negative bacterial pathogens were similar in both the survivor and non-survivor groups. *E. coli* was the leading pathogen; (4) Several risk factors were associated with mortality, including male gender, liver cirrhosis, malignancy, and septic shock. The increased levels of RDW, high serum lactate, and low serum albumin had the highest association with death probability, so we used them as parameters for revising scoring systems; (5) The original MEDS, revised MEDS, revised qSOFA, and revised NEWS were valuable tools for predicting the mortality risk in patients with afebrile bacteremia caused by monomicrobial GNB.

The reported literature described afebrile bacteremia as having a distinct presentation in the elderly and patients with an immunosuppressed status [5,6,7,8,9]. Yo et al. further reported that the oldest group (age ≥ 85 years) and solid malignancy were the comorbidities that were most apt to obtain afebrile bacteremia [9]. In this study, we also found that afebrile bacteremia of monomicrobial GNB most frequently occurred in those aged ≥75 years (42.66%, 256 of 600) and in patients with malignancy (53.83%, 323 of 600). We found a certain proportion of adult patients aged between 18 and 64 years old (36.66%, 220 of 600) in this study. Our study revealed a higher mortality rate of 50.17% in patients with afebrile bacteremia of monomicrobial GNB. In the subgroup of patients with liver cirrhosis, we found an extremely high mortality rate of up to 68.52%.

Yo et al. reported that *E. coli* infection was an independent negative predictor of afebrile bacteremia [9]. However, *E. coli* is the predominant causative pathogen (42.83%, 257 of 600) in this series of patients with afebrile bacteremia of monomicrobial GNB. The bacteriology was similar in both groups and other gram-negative pathogens, including *Klebsiella* spp. (20%), *Salmonella* spp. (6%), and *Pseudomonas* spp. (5%) were present. Previous research on GNB reported that *E. coli* is the most common pathogen in patients with community-acquired bacteremia, occurring in 26.6% of patients, and this is the second most common bacteria within hospital-acquired infection, occurring in 21.3% of patients. *Klebsiella pneumoniae* is the third most common pathogen for both community- and hospital-acquired bacteremia, responsible for 7.2% and 8.8% of patients, respectively. *Pseudomonas aeruginosa* is the fourth most prevalent pathogen of hospital-acquired bacteremia (7.4% of patients) and ranked the fifth most common pathogen causing community-acquired bacteremia (7.3% of patients) [17,18,19]. In our series of patients, specific causative pathogens, such as *E. coli*, *Klebsiella* spp., *Salmonella* spp., and *Pseudomonas* spp., were not associated with increased mortality in patients with afebrile bacteremia caused by monomicrobial GNB.

The following factors were associated with in-hospital mortality, including male gender, liver cirrhosis, malignancy, and septic shock in this study of patients with afebrile bacteremia of monomicrobial GNB. Our findings were consistent with previous studies that reported a mortality risk associated with sepsis. At an older age, immunosuppressive diseases, and DM are well-established risk factors relating to a patient’s susceptibility to be infected and becoming victim to different organ failures [20]. Some epidemiological studies have revealed a lower prevalence of sepsis in women than in men [21,22]. However, the evidence on how gender influences clinical outcomes in sepsis was changeable from previous retrospective studies, and there is no precise evidence on how gender impacts the outcomes in sepsis [23,24]. In this series of patients, we found that men had significantly higher in-hospital mortality rates than women (67.10% vs. 32.89%, *p* < 0.001) among those with afebrile bacteremia of monomicrobial GNB.

Patients with liver cirrhosis, complicating the bacterial infection, frequently have atypical manifestations, such as an afebrile state [25]. Chen et al. described that ED patients with afebrile bacteremia showed a higher rate of inappropriate antibiotic administration. They also had a higher 30-day mortality rate than the febrile group (40% vs. 18.4%) [26]. We found that cirrhotic patients with afebrile bacteremia of microbial GNB had an extremely high mortality rate of 68.52% (74 of 108). On the univariate logistic regression modeling, liver cirrhosis associated with a higher OR of2.541 (*p* < 0.001) was a positive predictor of the mortality risk.

In this study, hyperlipidemic patients with afebrile bacteremia of monomicrobial GNB had a lower OR of 0.458 (*p* < 0.001) associated with death. In an animal study, Morin et al. reported that higher high-density lipoprotein (HDL) cholesterol levels were related to a lower mortality risk of sepsis [27]. Furthermore, in a prospective cohort study, Chien et al. found that a lower low-density lipoprotein (LDL) cholesterol level on day 1 of severe sepsis had a higher mortality rate and grave outcomes [28]. Hyperlipidemia may be a protective factor in patients with afebrile bacteremia of monomicrobial GNB. However, lipid profiles, including HDL and LDL of cholesterol, were not routinely assessed in the ED. Further studies are required to gain evidence on how hyperlipidemia influences the clinical outcomes of bacteremia.

The authors developed various easy-to-apply scoring systems based on clinical parameters, in order to aid physicians in identifying potentially critical conditions early and quickly stratify patients in the ED or intensive care units (ICU) [29,30,31,32,33]. Shapiro et al. first developed the MEDS score in 2003, including nine parameters (age, nursing home residence, terminal disease, respiratory difficulty, lower respiratory infection, septic shock, platelet, band proportion, and altered mental status). Due to its ready availability, ED clinicians could use the MEDS score to evaluate the mortality risk during the patient’s presentation [29]. This score accurately predicts mortality in ED patients with suspected infection [34,35]. Smith et al. first published the NEWS in 2012, which demonstrated outstanding ability and a high maximum AUC of the ROC in predicting risk in patients with cardiac arrest, unplanned ICU admission, or death within 24 h [30,36]. Physicians performed the NEWS, including body temperature, heart rate, respiratory rate, systolic blood pressure, oxygen saturation, the necessity of oxygen supply, and consciousness level, to measure the scores during the ED course. As a rapid and more simplified ED sepsis screening tool, the qSOFA consists of three items, including altered mental status (GCS < 15), respiratory rate ≥ 22/min, and systolic blood pressure ≤ 100 mmHg [4,35]. Although the qSOFA may be a tool to predict sepsis-related mortality, some studies suggested it performs poorly in predicting severe sepsis and mortality [37,38,39].

In this single-center retrospective study, we found that the non-survivors had higher scores of the original MEDS, NEWS, MEWS, qSOFA, and REMS than the survivors, which were associated with a higher risk of death on univariate logistic regression analyses. The original MEDS showed the best performance in predicting the mortality risk of adult patients with afebrile bacteremia caused by monomicrobial GNB. The AUC of the ROC of MEDS was 0.773 at a cut-off point of 11, with a sensitivity of 75% and a specificity of 68%. The qSOFA and NEWS demonstrated an acceptable performance in predicting the mortality risk, with the AUCs of the ROC of 0.633 and 0.572, respectively (Figure 1).

Based on the univariate and multivariate analytic results of laboratory variables, we found that increased levels of RDW, high serum lactate, and low serum albumin were highly associated with the mortality rate (Table 5 and Table 6). Some studies suggested that RDW could be a risk variable in patients with sepsis and septic shock [40,41]. Serum lactate, acidosis, and hypoalbuminemia had clarified positive associations with the mortality rate in severe sepsis cases [37,42,43,44]. All these parameters were in the form of readily available data within 2 h during the ED workup. Therefore, we applied revisions of the original MEDS, qSOFA, and NEWS by adding scores of RDW (RDW > 16.5%, score = 1; RDW > 19.9%, score = 2), albumin (albumin < 3.0 g/dL, score = 1; albumin < 2.4 g/dL, score = 2), and lactate (lactate > 21 mg/dL, score = 1; lactate > 48 mg/dL, score = 2) to get a better performance than that of the original scoring systems. The revised MEDS (cut-off point of 11) remained the best performance in predicting mortality, with an AUC of the ROC of 0.797, a sensitivity of 87%, and a specificity of 60%. The revised qSOFA (cut-off point of 3) and NEWS (cut-off point of 6) simultaneously showed a superior performance than the original systems, with the AUCs of the ROC of 0.719 and 0.694, respectively. Our findings suggested that the original MEDS, revised MEDS, revised qSOFA, and revised NEWS were valuable tools for predicting the mortality risk in patients with afebrile bacteremia of monomicrobial GNB.

## 5. Limitations

Several limitations are inherent to the present study. First, this is a single-center study with a retrospective design, in which clinical data and variables might not represent the complete characteristics of the disease. Second, there is no objective body temperature measurement for all enrolled patients before presenting to the ED. An exclusive reliance on the patients’ or caregivers’ subjective fever history may inevitably lead to recall bias. Third, this study lacks detailed information on admission source (community, nursing home, or hospital) and site of infection (e.g., respiratory, abdominal, skin/soft tissue, or urinary), so we could not find out whether some of these factors may result in different clinical outcomes. Fourth, the timing and appropriateness of antibiotic therapy play a crucial role in the prognosis of bacteremia management. Due to the lack of records on detailed antibiotic therapy in this study, we could not further analyze how those treatments may influence the clinical outcomes.

## 6. Conclusions

In the present study, we found that male gender, liver cirrhosis, malignancy, and septic shock were risk factors associated with the in-hospital mortality of adult patients with afebrile bacteremia caused by monomicrobial GNB. We have also shown that adding RDW, serum albumin, and lactate scores promotes the risk-predicting performance of MEDS, qSOFA, and NEWS on short-term outcomes of monomicrobial GNB afebrile bacteremia. The original MEDS, revised MEDS, revised qSOFA, and revised NEWS were valuable tools to predict the mortality risk in patients with afebrile bacteremia of monomicrobial GNB. We suggested that ED clinicians should explore patients with the risk factors mentioned above for possible severe infection, even in the absence of fever, and initiate hemodynamic support and early adequate antibiotic therapy in patients with higher scores of the original MEDS (≥11), revised MEDS (≥11), revised NEWS (≥6), and revised qSOFA (≥3).

## Figures and Tables

**Table 1:** Demographic characteristics, comorbidities, and outcomes of 600 adult patients with afebrile bacteremia of monomicrobial GNB.

General Data	All (*n* = 600)	Survivors (*n* = 299)	Non-Survivors (*n* = 301)	*p*-Value
Male ^f^	358 (59.66%)	156 (52.17%)	202 (67.1%)	<0.001 **
Age	69.59 ± 15.38	68.97 ± 15.43	70.20 ± 15.34	0.490
18–40	29 (4.83%)	21 (7.02%)	8 (2.65%)	0.021 *
41–64	191 (31.83%)	86 (28.76%)	105 (34.88%)	0.128
65–74	124 (20.66%)	67 (22.4%)	57 (18.93%)	0.343
≥75	256 (42.66%)	125 (41.8%)	131 (43.52%)	0.732
DNR	357 (59.5%)	90 (30.1%)	267 (88.7%)	<0.001 **
Vital signs				
SBP	115.12 ± 29.18	119.03 ± 29.70	111.24 ± 28.16	0.005 **
DBP	68.75 ± 18.43	70.28 ± 17.16	67.23 ± 19.52	0.021 *
MAP	84.21 ± 20.46	86.53 ± 19.88	81.90 ± 20.79	0.009 **
HR	95.76 ± 21.50	93.14 ± 21.31	98.36 ± 21.40	0.001 **
RR	19.21 ± 3.51	18.87 ± 2.72	19.54 ± 4.12	0.438
BT	36.33 ± 0.80	36.44 ± 0.76	36.23 ± 0.82	0.001 **
GCS	14.04 ± 2.65	14.15 ± 2.53	13.93 ± 2.76	0.142
SpO_2_	96.72 ± 4.23	97.31 ± 2.26	96.19 ± 5.36	0.935
Comorbidities				
Malignant tumor ^f^	323 (53.83%)	131 (43.81%)	192 (63.78%)	<0.001 **
GI disease ^f^	272 (45.33%)	127 (42.47%)	145 (48.17%)	0.187
Chronic renal disease ^f^	195 (32.5%)	94 (31.43%)	101 (33.55%)	0.641
DM ^f^	181 (30.16%)	94 (31.43%)	87 (28.9%)	0.557
Hyperlipidemia ^f^	158 (26.33%)	101 (33.77%)	57 (18.93%)	<0.001 **
Biliary tract disease ^f^	133 (22.16%)	71 (23.74%)	62 (20.59%)	0.407
COPD ^f^	114 (19%)	56 (18.72%)	58 (19.26%)	0.949
Liver cirrhosis ^f^	108 (18%)	34 (11.37%)	74 (24.58%)	<0.001 **
Clinical course				
ICU admission ^f^	219 (36.5%)	85 (28.42%)	134 (44.51%)	<0.001 **
Respiratory failure ^f^	112 (18.66%)	42 (14.04%)	70 (23.25%)	0.005 **
Total stay (day)	19.30 ± 19.94	22.56 ± 19.47	16.00 ± 19.89	<0.001 **
ICU stay (day)	13.01 ± 13.89	15.61 ± 13.27	11.36 ± 14.06	<0.001 **
Treatment				
O_2_ supply ^f^	366 (61%)	170 (56.85%)	196 (65.11%)	0.047 *
Vasopressor ^f^	293 (48.83%)	111 (37.12%)	182 (60.46%)	<0.001 **
Septic shock ^f^	92 (15.33%)	32 (10.7%)	60 (19.93%)	0.002 **

Chi-Square test. ^f^ Fisher’s Exact test. Mann–Whitney U-test. * *p* < 0.05, ** *p* < 0.01, Statistically significant. Continuous data are expressed as mean ± SD. Categorical data are expressed as numbers and percentages. Abbreviations: BT, Body temperature; COPD, Chronic obstructive pulmonary disease; DBP, Diastolic blood pressure; DM, Diabetes mellitus; DNR, Do not resuscitate; GCS, Glasgow coma scale; GI: Gastrointestinal; HR, Heart rate; ICU, intensive care unit; MAP, Mean arterial pressure; RR, Respiratory rate; and SBP, Systolic blood pressure.

**Table 2:** Laboratory data of 600 adult patients with afebrile bacteremia of monomicrobial GNB.

Laboratory Data	All (*n* = 600)	Survivors (*n* = 299)	Non-Survivors (*n* = 301)	*p*-Value
Blood cell counts				
WBC (×10^3^ counts/mm^3^)	14808.08 ± 10137.33	15490.23 ± 8540.97	14130.47 ± 11480.52	0.003 **
Hemoglobin (g/dL)	10.87 ± 2.60	11.28 ± 2.65	10.45 ± 2.49	<0.001 **
Platelet (×10^3^ counts/mm^3^)	175.30 ± 117.76	194.01 ± 119.91	156.70 ± 112.75	<0.001 **
RDW (%)	16.22 ± 3.05	15.31 ± 2.63	17.12 ± 3.18	<0.001 **
Biochemistry				
Albumin (g/dL)	2.69 ± 0.62	2.83 ± 0.61	2.56 ± 0.60	<0.001 **
Total bilirubin (mg/dL)	2.74 ± 5.18	1.81 ± 3.16	3.66 ± 6.45	<0.001 **
BUN (mg/dL)	54.52 ± 42.22	48.41 ± 38.91	60.61 ± 44.52	<0.001 **
Cr (mg/dL)	2.68 ± 2.32	2.55 ± 2.36	2.81 ± 2.28	0.026 *
BUN/Cr	24.56 ± 15.32	22.76 ± 14.49	26.36 ± 15.94	0.001 **
CRP (mg/dL)	16.17 ± 11.41	16.37 ± 11.92	15.96 ± 10.87	0.855
Lactate (mg/dL)	35.68 ± 33.76	28.24 ± 28.24	42.82 ± 36.99	<0.001 **
Glucose (mg/dL)	186.80 ± 146.51	195.37 ± 137.02	178.29 ± 155.14	0.013 *
Coagulation profile				
PT (s)	13.73 ± 5.96	12.16 ± 2.79	15.30 ± 7.63	<0.001 **
APTT (s)	35.29 ± 13.37	31.60 ± 7.21	38.89 ± 16.63	<0.001 **
Arterial blood gas				
pH	7.35 ± 0.11	7.37 ± 0.09	7.34 ± 0.12	0.013 *
HCO_3_^−^ (mmol)	20.10 ± 6.58	20.91 ± 6.14	19.45 ± 6.86	0.023 *

Chi-Square test. Mann–Whitney U test. * *p* < 0.05, ** *p* < 0.01, Statistically significant. Continuous data are expressed as mean ± SD. Categorical data are expressed as numbers and percentages. Abbreviations: APTT, Activated partial prothrombin time; BUN, Blood urea nitrogen; CRP, C-reactive protein; Cr, Creatinine; PT, Prothrombin time; and RDW, red cell distribution width.

**Table 3:** Scoring systems for predicting the mortality risk of 600 adult patients with afebrile bacteremia of monomicrobial GNB.

Scoring Systems	All (*n* = 600)	Survivors (*n* = 299)	Non-Survivors (*n* = 301)	*p*-Value
MEDS	10.26 ± 5.00	7.92 ± 4.97	12.63 ± 3.77	<0.001 **
NEWS	4.83 ± 3.21	4.11 ± 3.07	5.54 ± 3.18	<0.001 **
MEWS	1.97 ± 2.18	1.64 ± 1.97	2.31 ± 2.33	<0.001 **
qSOFA	0.66 ± 0.77	0.55 ± 0.73	0.76 ± 0.79	<0.001 **
REMS	6.38 ± 2.89	6.07 ± 2.67	6.68 ± 3.07	0.030 *

* *p* < 0.05, ** *p* < 0.01, Statistically significant. Abbreviations: MEDS, Mortality in Emergency Department Sepsis; NEWS, National Early Warning Score; MEWS, Modified Early Warning Score; qSOFA, quick Sequential Organ Failure Assessment; and REMS, Rapid Emergency Medicine Score.

**Table 4:** Microbiological findings of 600 adult patients with afebrile bacteremia of monomicrobial GNB.

Microorganism	All (*n* = 600)	Survivors (*n* = 299)	Non-Survivors (*n* = 301)	*p*-Value
*Escherichia coli*	257 (42.83%)	137 (45.81%)	120 (39.86%)	0.164
*Klebsiella* spp.	120 (20%)	51 (17.05%)	69 (22.92%)	0.090
*Salmonella* spp.	36 (6%)	22 (7.35%)	14 (4.65%)	0.221
*Pseudomonas* spp.	30 (5%)	14 (4.68%)	16 (5.31%)	0.866
*Proteus* spp.	27 (4.5%)	14 (4.68%)	13 (4.31%)	0.986

*p* < 0.05, Statistically significant. Abbreviations: spp., species.

**Table 5:** Odds ratio and 95% confidence interval of univariate logistic analyses for primary outcome.

Characteristics	Odds Ratio	95% Confidence Interval	*p*-Value
Age (years)	1.005	0.995–1.016	0.326
Male	0.535	0.384–0.744	<0.001 **
Clinical course			
Septic shock	2.077	1.307–3.301	0.002 **
ICU admission	2.020	1.440–2.835	<0.001 **
Vital signs			
SBP (mmHg)	0.991	0.985–0.996	0.001 **
MAP (mmHg)	0.989	0.981–0.997	0.006 **
GCS	0.969	0.912–1.030	0.317
Clinical treatment			
Oxygen supply	1.416	1.019–1.969	0.038 *
Vasopressor	2.590	1.864–3.061	<0.001 **
Comorbidities			
Cardiovascular disease	0.777	0.503–1.200	0.255
DM	0.887	0.625–1.257	0.499
CKD	1.101	0.782–1.550	0.580
Hyperlipidemia	0.458	0.315–0.666	<0.001 **
Liver cirrhosis	2.541	1.631–3.957	<0.001 **
Malignant tumor	2.259	1.628–3.135	<0.001 **
Laboratory data			
White blood cell (counts/μL)	1.000	1.000–1.000	0.015 *
Hemoglobin (g/dL)	0.881	0.827–0.939	<0.001 **
Platelet (×10^3^ counts/μL)	0.997	0.996–0.999	<0.001 **
Red cell distribution width (RDW)	1.260	1.179–1.347	<0.001 **
Albumin (g/dL)	0.468	0.346–0.632	<0.001 **
Total bilirubin (mg/dL)	1.095	1.046–1.146	<0.001 **
BUN (mg/dL)	1.007	1.003–1.011	0.001 **
Cr (mg/dL)	1.049	0.978–1.125	0.177
BUN/Cr	1.017	1.005–1.029	0.005 **
C-reactive protein (mg/dL)	0.997	0.983–1.011	0.672
Lactate (mg/dL)	1.016	1.009–1.022	<0.001 **
PT (s)	1.273	1.183–1.369	<0.001 **
APTT (s)	1.068	1.044–1.092	<0.001 **
pH	0.062	0.010–0.383	0.003 **
HCO_3_^−^ (mmol/L)	0.966	0.939–0.994	0.018 *
Scoring systems			
MEDS	1.264	1.209–1.321	<0.001 **
NEWS	1.160	1.099–1.225	<0.001 **
MEWS	1.159	1.072–1.252	<0.001 **
qSOFA	1.430	1.153–1.774	0.001 **
REMS	1.078	1.019–1.142	0.009 **

* *p* < 0.05, ** *p* < 0.01, Statistically significant. Abbreviations: APTT, Activated partial prothrombin time; BUN, Blood urea nitrogen; CKD, Chronic kidney disease; Cr, Creatinine; DM, Diabetes mellitus; GCS, Glasgow coma scale; ICU, Intensive care unit; MAP, Mean arterial pressure; PT, Prothrombintime; SBP, Systolic blood pressure; MEDS, Mortality in Emergency Department Sepsis; NEWS, National Early Warning Score; MEWS, Modified Early Warning Score; qSOFA, quick Sequential Organ Failure Assessment; and REMS, Rapid Emergency Medicine Score.

**Table 6:** Odds ratio and 95% confidence interval of univariate and multivariate logistic regression analyses of laboratory variables for primary outcome.

	Univariate			Multivariate		
Variables	OR	95% CI	*p*-Value	OR	95% CI	*p*-Value
Platelet	0.997	0.996–0.999	<0.001 **	0.999	0.997–1.000	0.146
RDW	1.260	1.179–1.347	<0.001 **	1.194	1.098–1.299	<0.001 **
Albumin	0.468	0.346–0.632	<0.001 **	0.643	0.441–0.938	0.022 *
BUN	1.007	1.003–1.011	<0.001 **	1.002	0.997–1.007	0.457
BUN/Cr	1.017	1.005–1.029	0.005 **	1.013	0.998–1.028	0.081
Lactate	1.016	1.009–1.022	<0.001 **	1.009	1.001–1.017	0.021 *
pH	0.062	0.010–0.383	0.003 **	0.122	0.012–1.287	0.080

* *p* < 0.05, ** *p* < 0.01, Statistically significant. Abbreviations: CI, Confidence interval; BUN, Blood urea nitrogen; Cr, Creatinine; OR, Odds ratio; and RDW, Red cell distribution width.

**Table 7:** The AUC of the ROC, cut-off point (COP), sensitivity specificity, positive predictive value (PPV), negative predictive value (NPV), accuracy, and standard error (SE) of the original MEDS, NEWS, and qSOFA to predict the mortality risk.

Scores	AUC	COP	Sensitivity	Specificity	PPV	NPV	Accuracy	SE	*p*-Value
MEDS	0.773	11	75%	68%	70%	73%	71%	0.019	<0.001 **
qSOFA	0.633	5	61%	62%	62%	61%	61%	0.023	<0.001 **
NEWS	0.572	1	57%	58%	57%	57%	57%	0.023	0.002 **

** *p* < 0.01, Statistically significant. Abbreviations: AUC, Area under the curve; COP, Cut-off point; MEDS, Mortality in Emergency Department Sepsis; NEWS, National Early Warning Score; qSOFA, quick Sequential Organ Failure Assessment; NPV, Negative predictive value; PPV, Positive predictive value; ROC, Receiver operating characteristic curve; and SE, Standard error.

**Table 8:** The AUC of the ROC, cut-off point (COP), sensitivity specificity, positive predictive value (PPV), negative predictive value (NPV), accuracy, and standard error (SE) of the revised MEDS, revised NEWS, and revised qSOFA to predict the mortality risk.

Scores	AUC	COP	Sensitivity	Specificity	PPV	NPV	Accuracy	SE	*p*-Value
R-MEDS	0.797	11	87%	60%	68%	82%	73%	0.018	<0.001 **
R-qSOFA	0.719	3	67%	68%	67%	67%	67%	0.021	<0.001 **
R-NEWS	0.694	6	74%	56%	63%	68%	65%	0.021	<0.001 **

** *p* < 0.01, Statistically significant. Abbreviations: AUC, Area under the curve; COP, Cut-off point; R-MEDS, Revised Mortality in Emergency Department Sepsis; R-qSOFA, Revised quick Sequential Organ Failure Assessment; R-NEWS, Revised National Early Warning Score; NPV, Negative predictive value; PPV, Positive predictive value; ROC, Receiver operating characteristic curve; and SE, Standard error.

## Data Availability

The data are available upon reasonable request to the corresponding author. Readers can access the data and material supporting the study’s conclusions by contacting Sung-Yuan Hu at song9168@pie.com.tw.

## Supplementary Materials

The following supporting information can be downloaded at: https://www.mdpi.com/article/10.3390/diagnostics14090869/s1. Table S1: Scoring systems [29,30,37,45,46].
