# Supplementary material for: Risk Factors and Scoring Systems to Predict the Mortality Risk of Afebrile Adult Patients with Monomicrobial Gram-Negative Bacteremia: A 10-Year Observational Study in the Emergency Department
Source: Diagnostics (Basel). 2024 Apr 23;14(9):869. doi: 10.3390/diagnostics14090869 (PMC11083546; doi:10.3390/diagnostics14090869)
Supplement: Supplementary file 1 [file diagnostics-14-00869-s001.zip › diagnostics-2947897-supplementary.pdf]

Table S1: scoring systems

Mortality in emergency department sepsis (MEDS) score [29]

Mortality in emergency department sepsis (MEDS) score		Points
1.	Terminal illness with possible death in 1 month	6
2.	Hypoxia or tachypnea	3
3.	Shock from sepsis	3
4.	Platelet count below 150,000	3
5.	Granulocytic bands >5% of white blood cells	3
6.	Patient older than 65 years old	3
7.	Lower respiratory infection	2
8.	Patient is from a nursing home	2
9.	Mental status is altered	2

National Early Warning Score [30]

Physiological parameters	3	2	1	0	1	2	3
Respiration Rate (breaths per minute)	≤8		9–11	12–20		21–24	≥25
SpO2 (%)	≤91	92–93	94–95	≥96			
Any supplemental oxygen		Yes		No			
Temperature (°C)	≤35.0		35.1–36.0	36.1–38.0	38.1–39.0	≥39.1	
Systolic BP (mmHg)	≤90	91–100	101–110	111–119			≥220
Heart/pulse rate (beats per minute)	≤40		41–50	51–90	91–110	111–130	≥131
Level of consciousness (AVPU system)				A			V, P or U

Modified Early Warning Score [45]

Points	3	2	1	0	1	2	3
Temperature(°C)		35 or less		35.1–38.4		38.5 or higher	
Heart rate (bpm)		39 or less	40–50	51–100	101–110	111–129	130 or higher
Systolic blood pressure (mmHg)	70 or less	71–80	81–100	101–199		200 or higher	
Respiratory rate		8 or less	9	10–18	19–20	21–29	30 or higher
AVPU score				A	V	P	U

Rapid Emergency Medicine Score [46]

REMS scoring system	Score						
Variables	0	+1	+2	+3	+4	+5	+6
Age (years)	<45		45–54	55–64		65–74	>74
Mean arterial pressure (mmHg)	70–109		110–129<br>50–69	130–159	>159<br>≤49		
Heart rate (bpm)	70–109		110–139<br>55–69	140–179<br>40–54	>179<br>≤39		
Respiratory rate (breaths/min)	12–24	25–34<br>10–11	6–9	35–49	>49<br>≤5		
O2 saturation (%)	>89	86–89		75–85	<75		
Glasgow Coma Scale	14or 15	11–13	8–10	5–7	3 or 4		

quick Sequential Organ Failure Score [37]

qSOFA	Point
Respiratory Rate >22	1
Systolic Blood Pressure <100	1
Glasgow Coma Scale <14	1
